# To tell a morphologically complex tale: investigating the story-telling abilities of children and adults with low literacy skills

**DOI:** 10.1007/s11145-015-9560-5

**Published:** 2015-04-01

**Authors:** Katherine S. Binder, Brooke Magnus, Cheryl Lee, Nicole Gilbert Cote

**Affiliations:** 1 Psychology and Education Department, Mount Holyoke College, 50 College St., South Hadley, MA 01075 USA; 2 University of North Carolina, Chapel Hill, Chapel Hill, NC USA

**Keywords:** Morphological awareness, Morphology and writing, Adults with low literacy, Adult literacy, Morphologically complex words, Spelling, vocabulary and morphological awareness

## Abstract

This study examined differences between adults with low literacy skills and typically achieving children, who were matched on decoding ability, on their production of morphologically complex words (MC) in oral and written stories. In addition, we collected data on their morphological awareness, spelling, and vocabulary skills. Both adults and children were more likely to produce MC words in their oral stories compared to their written stories. While children were much more skilled at using –*ed* forms to produce past tense verbs than adults, adults were more likely to add –*s* to a verb and to produce contractions compared to children. Children and adults were comparable in pluralizing words, adding –*ing* to verbs, and producing derived MC words. For all of the literacy measures (morphological awareness, spelling, and vocabulary) adults always outperformed children. Thus, while adults were stronger in morphological awareness, spelling, and vocabulary, those skills did not seem to aid in the growth of explicitly applying morphological knowledge in the story-telling tasks.

## Introduction

According to the results of the recent Program for the International Assessment of Adult Competencies [PIAAC, American Institutes for Research (AIR) PIAAC, 2014; NCES, 2014], adults in the US performed significantly below the international averages for literacy, numeracy, and problem-solving. Further, the results showed that 1 in 6 adults in the US reads at an elementary level. There are many potential ramifications of low literacy that include, but are not limited to, vital social, health, and economic issues. Recognizing the need for appropriate and effective literacy programs for adults and children alike, researchers have tried to untie the knots that affect literacy achievement by isolating and investigating the contributions of cognitive and linguistic factors. To date, progress has been made on understanding some of the similarities and differences in phonological and orthographic processes between adults and children (Greenberg, Ehri, & Perin, 1997, 2002; Thompkins & Binder, 2003), but the role of morphological awareness for adults with low literacy skills remains largely unstudied. Furthermore, the role of morphological awareness in the development of writing abilities for these adults has received even less attention. Since writing is a difficult task, morphological awareness could facilitate this process by providing the tools needed to access a broader vocabulary while writing, making it easier to find the words in long-term memory that will convey their ideas (Green et al., 2003). Given the substantial need for evidence-based instruction in adult literacy populations, the purpose of the current study was to examine the use of morphologically complex (MC) words in oral and written stories for both adults with low literacy skills and children who were matched with the adults on decoding skills. In addition, we assessed the development of their morphological awareness, spelling, and vocabulary skills.

Morphemes are the smallest units of meaning in a language, and are comprised of roots and affixes. The most studied MC words are inflectional and derivational words (Anglin, 1993; Brown, 1973). Inflectional morphemes are suffixes that preserve the root word but change its tense as in *cook* to *cooked* or quantity as in *flower* to *flowers* (Katamba, 1993). Research on oral language development indicates that preschool-aged children understand and produce simple inflectional morphemes implicitly—without conscious awareness of grammatical rules (Carlisle, 2003). Awareness of inflectional morphology continues to develop through the elementary school years (Bryant, Nunes, & Bindman, 1997; Rubin, Patterson, & Kantor, 1991). Derivational morphemes, on the other hand, can change the meaning of a word such as adding *ir*- to *regular* to form *irregular*, as well as the part of speech by adding –*ly* to *slow* to form *slowly* (Katamba, 1993). This is generally the area of morphology with which children have the most difficulty, and is often the last to begin to develop (Anglin, 1993; Berko, 1958; Clark, 1982; Wysocki & Jenkins, 1987). While inflectional morphological awareness shows the most growth in grades one through four, derivational morphological awareness shows increased growth after the fourth grade (Anglin, 1993; Berninger, Abbott, Nagy, & Carlisle, 2010; Carlisle, 2000, 2003; Derwig & Baker, 1979; Freyd & Baron, 1982; Kruk & Bergman, 2013; Tong, Deacon, Kirby, Cain, & Parrila, 2011).

When someone is able to manipulate and decompose words into their constituent morphemes, he or she is said to have morphological awareness (Fowler & Liberman, 1995; Katamba, 1993). Morphological awareness has the potential to assist literacy development in a number of ways. It makes readers more aware of the system of writing, allows them to read and spell relatively long words, serves as a tool for breaking words down into chunks, creates more links between words in the mental lexicon, and increases analytical skills by requiring readers to understand each constituent of a word to arrive at the correct definition (Carlisle, 2000; Nagy, Berninger, Abbott, Vaughan, & Vermeulen, 2003).

To date, most of the research on morphological awareness has focused on its development in children. This research suggests that children use their knowledge of base morphemes to assist in the recognition of words (Carlisle, 2000; Carlisle & Stone, 2005; Singson, Mahony, & Mann, 2000). During the early elementary school grades, skills related to morphological awareness and phonological awareness tend to overlap (Jarmulowicz, Taran, & Hay, 2007), but past this point, morphological awareness becomes increasingly important (Nagy, Berninger, & Abbott, 2006). More recently, To, Tighe, and Binder (2014) established that morphological awareness explained variance in word reading for adults with low literacy skills. After controlling for pseudo-word decoding, morphological awareness explained an additional 5 % of the variance for this group. Thus, this group of developing readers also relies on this form of information.

Researchers have also examined the relationship between morphological awareness and spelling. Deacon and Bryant (2006) found evidence that children use morphological knowledge as a spelling strategy, at least by the age of seven. Singson et al. (2000) assessed children in grades three through six and found that a greater knowledge of derivational morphology was associated with a greater ability to correctly spell words. Nagy et al. (2006) studied the effects of morphological awareness on spelling and found that it made a unique significant contribution to spelling for students in grades four through nine. The relationship between morphological awareness and spelling has also been found for adults with low literacy skills (Fracasso, Bangs, & Binder, 2014), even after controlling for phonological decoding skills. Fracasso et al. found that morphological awareness accounted for an additional 5 % of the variance in spelling abilities, after controlling for pseudo-word decoding. However, while Talwar, Gilbert Cote, and Binder (2014) found a significant correlation between spelling abilities and morphological awareness (*r* = .46), morphological awareness was not a unique predictor in the regression analyses for spelling, after phonological abilities had been added. Other studies that have examined spelling errors demonstrate that adults with low literacy skills struggle with morphological markers in spelling. For example, adults with low literacy skills were more likely to omit (*chop* instead of *chopping)* or incorrectly substitute (*speaking* instead of *speaker*) affixes in MC words (Viise, 1996; Worthy & Viise, 1996). This pattern has been observed in both inflectional and derivational MC words. Based on these studies, it is clear that morphological awareness is related to spelling abilities.

Research with children has shown that morphological awareness is also related to vocabulary skills (Fowler & Liberman, 1995; Kuo & Anderson, 2006; McBride-Chang et al., 2008). Nagy and Anderson (1984) predicted that readers can understand the meanings of 60 % of the unfamiliar words they encounter by breaking the word down into its constituent morphemes. Having an awareness of the structure of words can assist in defining these unfamiliar words, as there is a significant correlation between morphological awareness and the ability to define words at certain grade levels (Carlisle, 2000). This relationship has been established for adults with low literacy skills, too (Fracasso et al., 2014; Herman, Gilbert Cote, Reilly, & Binder, 2013; Tighe & Schatschneider, 2014). In Fracasso et al., morphological awareness explained an additional 41 % of the variance in vocabulary after controlling for pseudo-word decoding, and in Herman et al., morphological awareness was a unique predictor in the model explaining variance in vocabulary abilities.

While several studies have investigated the contribution of morphological awareness in reading and spelling, fewer studies have examined morphological awareness in writing. To speak fluently, one must have an implicit understanding of morphological rules (Rubin et al., 1991). Writing requires the same implicit awareness as well as an explicit understanding of morphemes and orthographic properties of words. Rubin (1991) hypothesized that this explicit understanding of morphemes is what sets proficient writers apart from poor ones.

Carlisle (1996) administered a spontaneous writing task to non-learning-disabled and learning-disabled second and third graders, and she examined the frequency and accuracy of the use of MC words. She found that third grade students more often wrote a narrative that used the past tense, whereas second grade students more often wrote a present tense description of the picture. This distinction is important, because use of the past tense of regular verbs requires a greater knowledge of MC words, mainly the addition of the inflectional morpheme –*ed*. The number of students using past tense, as well as the number of students using compound words, increased from second to third grade. It is also worth noting that the second graders made more errors in using MC words than third graders. Finally, Carlisle found a significant correlation between a morphological awareness task and the accuracy of use of MC words in the spontaneous writing task (*r* = .54).

Green et al. (2003) examined differences in writing among third and fourth graders in a study that used a cross-sequential design, measuring students’ abilities in both the fall and spring. They instructed the students to write a 15-min narrative about a provided picture. Results indicated that the types of MC words (e.g., inflectional vs. derivational) used most frequently varied across age. Both third and fourth graders used more inflectional forms correctly than derivational forms. When students did use derivational forms, fourth graders used them more accurately than third graders. Interestingly, by the spring of the fourth grade, there was not such a large difference in accurate use of derivational and inflected forms in the children’s writing. They also found that the accuracy in the use of MC words predicted both reading and spelling abilities of third and fourth graders.

Rubin et al. (1991) assessed implicit and explicit morphological awareness in typically achieving second graders, language-learning-disabled second graders, and adult literacy learners. The morphological awareness scores of the adult literacy learners did not significantly differ from either second grade group, which was well below the expected score for adults on both the implicit and explicit morphological awareness tasks. Through a spontaneous writing task, Rubin et al. found that morphological errors in writing corresponded to measures of both implicit and explicit morphological awareness in oral language. Furthermore, the typically achieving second graders rarely made morphological errors. In contrast, the language-learning-disabled second graders and the adults both made significantly more errors in the spontaneous writing task than the typically achieving second graders. It appears that the adult group, despite having had much more exposure to both oral and written language than the typically achieving second graders, lacked not only greater knowledge of morphemes than the second graders; they also had more difficulty applying their morphological knowledge to writing. The results of this study suggest that maturation and exposure do not necessarily increase morphological awareness and while the deficits may not be as apparent in oral language, they are very apparent in written language. Thus, even if a person seems to possess oral morphological skills, it is still important to examine morphemes in writing to have a clearer understanding of overall morphological knowledge.

Clearly adults with low literacy skills have difficulties with written skills, but are those difficulties found in their oral language skills as well? Hall, Greenberg, Laures-Gore, and Pae (2014) found that adults with low literacy skills have deficient oral vocabulary skills. Similarly, Sticht (1982) found that the oral vocabulary skills of adults with low literacy skills were nearly two grade levels below their reading grade level. Further, Taylor, Greenberg, Laures-Gore, and Wise (2012) found that oral syntactical abilities were impaired for this group. Finally, Eme, Lacroix, and Almecija (2010) compared adults who had proficient literacy skills to adults with low literacy skills, and found that the adults with low literacy skills tended to produce oral narratives that were significantly less coherent. Thus, the weaknesses that are apparent in written skills are also apparent in tasks that tap oral abilities. However, there has not yet been a study that directly compared the performance of adults with low literacy skills on written and oral narrative tasks.

The purpose of the current study was to compare adults with low literacy skills to typically developing children who were matched with the adults on decoding skills. Participants completed two story-telling tasks—an oral and a written task. We compared the number of MC words that were used in the stories. Participants were also assessed on morphological awareness, spelling, and vocabulary skills. We addressed a number of research questions. First, we were interested in the types of MC words that were generated in the task. We hypothesized that more inflected forms would be generated than derived forms. Second, we wanted to explore whether the type of task or age group would be related to the production of MC words. That is, would participants generate more MC words in an oral story versus written task, and would children produce more MC words than adults with low literacy skills? Finally, we were interested in how other literacy skills (i.e., morphological awareness, spelling, and vocabulary) explained variance in the production of MC words.

## Method

### Participants

We collected data from adults who were enrolled in adult basic education (ABE) programs and children who were matched with the adults on decoding ability. The adult participants included 63 adults from urban ABE programs located in Western Massachusetts. In these programs, adult learners spend three hours a day, 5 days a week in the classroom. The majority of this time is spent with a classroom instructor. We recruited the adults from all levels of the ABE programs but not from classes specific to English language learners. The age of the participants ranged from 17 to 82 with a mean of 31.6 (*SD* = 13.3). The racial and ethnic background of the participants and primary language spoken at home was varied (see Table 1 for demographics). Participants received $15.00 as compensation for their time.

**Table 1:** Demographics (race/ethnicity and primary language) of participants

Demographic	Adult ABE students (%)	Typically developing children (%)
*Race*/*ethnicity*		
Hispanic	49	25
African-American	17	16
Caucasian	19	43
Biracial	6	11
Other	5	1
Unreported	3	3
*Primary language*		
English	60	78
English and Spanish	6	10
Spanish	19	10
Other	12	0
Unreported	3	3

In addition, we had 63 child participants. Participants had an average age of 9.5 (*SD* = 1.7), and these children were recruited from the 1st, 2nd, 3rd, and 5th grades. The majority of these children were recruited from the same communities that housed the ABE programs. As a measure of socioeconomic status, each school or program was asked to provide the number of students on reduced or free lunch programs. For three of the schools, the percentage of students in either of these programs ranged from 9 to 12 %. Data were not available for the remaining schools and programs. Similar to the adults, the racial background of the participants and primary language spoken at home varied (see Table 1 for demographics). For the children, parental consent was attained for each student prior to testing, and assent was obtained from the child. After each task, the participants received a small reward as compensation.

Since we were examining the participants’ abilities to produce words, either orally or in written form, we matched the groups on decoding ability, using the Word Attack sub-test of the Woodcock Reading Mastery Tests—Revised (Woodcock, 1987). Phonological awareness has been shown to predict important literacy gains including spelling and word reading (Vaessen & Blomert, 2013). Other work has shown that writers use phonological awareness alongside other skills such as morphological and grapheme awareness to produce written work (Shanahan, 2006). Furthermore, Ahmed, Wagner, and Lopez’s (2014) longitudinal work demonstrated the importance of decoding skills on spelling mastery, and suggests “that the ability to read words correctly may facilitate writing them correctly, via mastery of phoneme–grapheme relations that are learned through reading” (431). Thus, this appeared to be a good variable on which to match, Indeed, there were no differences on this measure between the groups, *t*(124) = −.26, *p* = .80; adults *M* = 20.2 and children *M* = 20.5.

### Materials

Participants were administered two storytelling tasks and a battery consisting of several tests that assess language abilities. The battery included tests of three morphological awareness assessments, spelling, and vocabulary. Standard testing procedures were followed for each of these tests.

#### Storytelling tasks

Two storytelling tasks were administered in which participants were instructed to tell orally or write a story about one of two pictures: a girl trying to catch a monkey who is hanging from the light in a messy living room and a boy running on a beach with his dog surrounded by other animals including an alligator, turtle, and dinosaurs. These pictures were taken from children’s books by Smee (1989) and Donnelly (1989), respectively. The stories were counterbalanced for both the picture and type of storytelling task. During the written storytelling task, the researcher administering the test instructed the participant to write a story about the picture. The participant had 5 min^1^ to write the story. The use of MC words was coded, as well as any corrections to MC words the participant made during a 1-min revision period.

The person administering the test instructed the participant to tell a story about the picture. Because writing a story takes considerably longer than telling an oral story, participants were given approximately 1½ min to tell their stories. Stories were tape recorded and later transcribed for coding. During pilot testing with the children, these timeframes produced comparable length passages. However, during the actual assessments, participants produced longer oral stories than written stories.

#### Morphological awareness

To assess morphological awareness, three tests were used. The first test, called the Test of Morphological Structure: Derivation, assessed knowledge of morphological structure and was used in Carlisle (2000). Specifically, it involved assessing the participant’s knowledge of derivational morphemes. All questions followed the same format. The person administering the test read a target word aloud. She then read a sentence but excluded one word from the sentence, which was replaced with the word “blank”. The tests were administered orally so those with reading difficulties would not be disadvantaged. The participant’s task was to produce the missing word by using his or her knowledge of derivational morphemes to change the target word to make it fit in the blank. An example is “Farm. My uncle is a _____.” In this scenario, the participant said “farmer” and received 1 point, or he or she incorrectly answered the question and received no point. The test was administered orally and the participant responded orally. Participants had a practice round with two sentences before beginning the task, which contained 33 sentences. Administration was discontinued when a participant answered six items incorrectly. Five new items were added to the assessment used in Carlisle (2000) in order to prevent a ceiling effect for the more advanced participants. A previous study reported a Cronbach α reliability coefficient for this task of .97 (Tighe & Binder, 2014).

The second test, called the Test of Morphological Structure: Production, assessed the participant’s ability to use derivational morphemes to decompose words. This test was similar to the first morphological test in structure, in that the participant was provided with a target word followed by an incomplete sentence. An example was “Driver. Children are too young to _____.” The participant said “drive” and received 1 point, or he or she incorrectly answered the question and received no point. Once again, the participant had a 2-sentence practice round before beginning the task, which also contained 33 sentences. This task was administered and responded to orally. Administration was discontinued when a participant answered six items incorrectly. Five new items were added to the assessment used in Carlisle (2000) in order to prevent a ceiling effect for the more advanced participants. In a previous study, this test had a reliability of .97 (Tighe & Binder, 2014).

The final test of morphological awareness, called the Suffix Choice Test, assessed a participant’s ability to manipulate morphemes using pseudowords (Berninger, Abbott, Billingsley, & Nagy, 2001; Berninger & Nagy, 1999). The participant was given a written form of the test, where he or she saw a sentence and four choices with which to fill in a blank. The person administering the test also read the sentence and four choices aloud to the participant, so this task did not depend on the participant’s ability to decode words. An example of an item on this task is “Our teacher taught us how to _____ long words”. For this item, the participant was provided with the following choices: *jittling, jittles, jittled*, and *jittle*. A response of “jittle” received 1 point; a response of one of the other answers received no point. This task consisted of 14 items. Administration was discontinued when a participant incorrectly answered six items. A previous study reported a Cronbach α reliability coefficient for this task of .84 (Tighe & Binder, 2014).

#### Spelling

Participants’ spelling abilities were assessed using the Wechsler Individual Achievement Test—Second Edition. This test assessed the participant’s ability to spell dictated words. The person administering the test read aloud one word at a time and provided a sentence for context. The participant wrote each word dictated on a sheet of paper provided by the researcher. The words increased in difficulty across the test. The participant received 1 point for each correct response. The reliability of this test ranges from .93 to .94, depending on the age group being tested (Weschler, 1991). The test was discontinued after a participant answered a total of six items incorrectly.

#### Vocabulary

Participants’ vocabulary was assessed using the Peabody Picture Vocabulary Test—Third Edition (Dunn & Dunn, 1997). During each round of this task, participants were presented with a card which had four pictures on it. The person administering the test dictated a stimulus word and instructed the participant to point to the picture that described that word. The word was not provided in the context of a sentence, and the participant was not told whether or not his or her response was correct. Each round of pictures increased in difficultly, and the test continued until the participant made eight or more errors on a particular set of items. Participants were provided with a practice round consisting of two questions before the actual test began. Adult participants and third graders started at item #73, fifth graders started at item #85 and first and second graders started at item #49, per test administration guidelines. The median reliability of this test is .94 (Dunn & Dunn, 1997).

### Procedure

For the children, the tasks were administered over a 2-day span with one session on each day. Each session lasted approximately 20 min. Session one included one storytelling task and half of the language tasks. Session two included another storytelling task and the remainder of the language tasks. For the adult participants, all tasks were presented in one session. The order of presentation was counterbalanced across the participants. Immediately following testing, the participants were asked to report demographic information, which included date of birth, racial background, and the language most frequently spoken at home. Participants were tested in a quiet location.

## Results

### Use of morphologically complex words in written and oral stories

We examined the use of MC words in story-telling, both in oral and written forms. The adults and children were matched on a pseudoword decoding measure.

#### Prevalence of use

Consistent with Carlisle (1996) and Green et al. (2003), we calculated the percentage of participants who used various morphological forms in their stories at least once. For inflected forms, we identified usage of the following forms: plural nouns –*s* and –*es,* progressive or continuous verbs –*ing*, past tense –*ed*, third person singular present verbs –*s*, and past particle verbs –*en*. We also coded derived MC words, compound words, and contractions. The percentages for each of these forms are presented in Table 2. Adults and children were fairly competent in using –*s* or –*es* for form plural nouns. Similarly, adults and children were quite competent in adding the –*ing* inflectional marker in both their oral and written stories. For using the past tense -*ed* marker, children were more likely to use a past tense verb than adults. Adults were more competent than children in adding –*s* for third person singular present tense verbs. Neither group was likely to add –*en* to make a past participle verb. Adults and children were likely to use derived forms in both written and oral narratives, and both groups were more likely to use compounds in their oral narratives compared to their written narratives. Adults were more likely to use contractions, especially in their oral narratives, than children.

**Table 2:** Percentage, mean number of MC forms, and SE of participants using different forms of morphologically complex words in oral and written stories

Form	Oral		Written	
	Adults	Children	Adults	Children
Inflected plural –*s* and –*es*	78.7 %	89.5 %	58.7 %	66.7 %
	2.10 (.24)	2.58 (.24)	1.48 (.22)	1.32 (.17)
Inflected verb –*ing*	93.4 %	96.5 %	77.8 %	71.4 %
	3.84 (.31)	3.58 (.32)	2.40 (.24)	1.59 (.19)
Inflected	49.2 %	78.9 %	47.6 %	77.8 %
Verb –*ed*	1.44 (.30)	2.82 (.33)	.90 (.15)	2.16 (.21)
Inflected verb –*s*	52.5 %	15.8 %	33.3 %	9.5 %
	1.28 (.27)	.33 (.12)	.43 (.09)	.29 (.16)
Inflected verb –*en*	8.2 %	1.8 %	3.2 %	3.2 %
	.08 (.03)	.02 (.03)	.07 (.04)	.04 (.04)
Derived	86.9 %	91.2 %	87.3 %	79.4 %
	3.92 (.45)	3.83 (.47)	2.41 (.29)	2.60 (.30)
Compounds	72.1 %	80.7 %	47.6 %	58.7 %
	1.74 (.26)	2.25 (.27)	.95 (.15)	.83 (.16)
Contractions	82 %	64.9 %	22.2 %	33.3 %
	3.53 (.39)	2.23 (.40)	.30 (.10)	.49 (.10)

#### Frequency of use: inflected forms

The first set of analyses illustrated the percentage of participants in each group using various forms of MC words. For those analyses, a participant only needed to have at least one correct item for each form. The next set of analyses considers the frequency of instances of the various forms. In these analyses, we only considered unique forms. That is, a participant could have used 5 different verbs with –*ed* added to them, or a participant could have repeated the same verb with an –*ed* 5 times. We believe these two situations reflect differences in knowledge, so we wanted to analyze the number of unique instances.

**Fig. 1:**
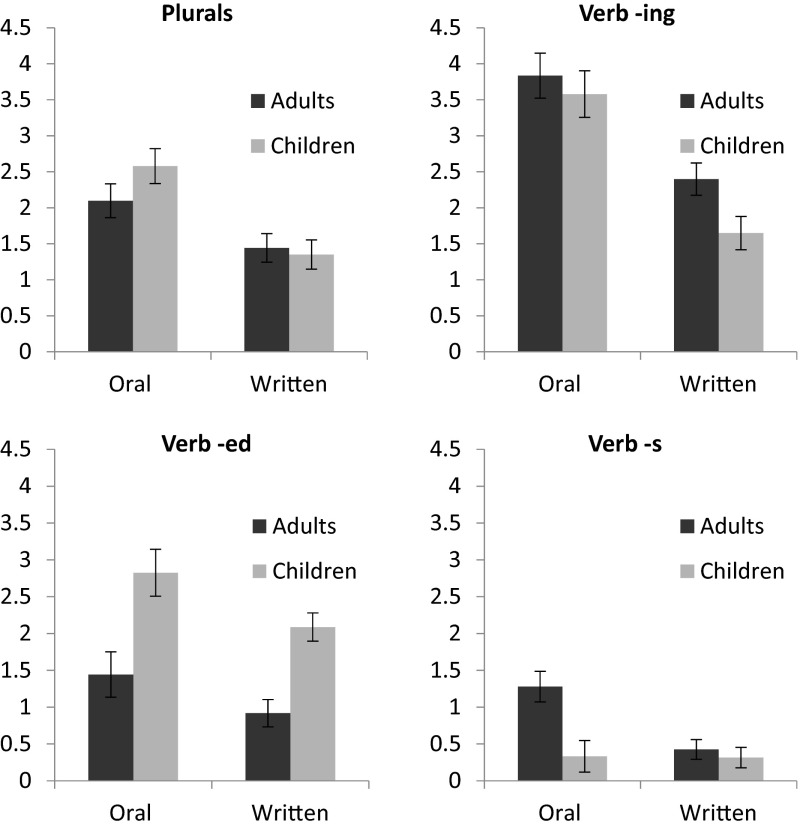
Mean (+/−SEM) number of inflected MC word types offered by adults and children across task

First, we conducted analyses using the various inflected forms. We conducted a 2 (Age: Adult vs. Child) × 2 (Task: Oral vs. Written) × 4 (Form: plural, -*ing*, -*ed*, -*s* 2) mixed measures ANOVA in which Age was the independent groups variable and Task and Form were repeated measures. The dependent variable was the number of times the participant uniquely used a particular inflected form. For all ANOVAs, significant effects were followed up using post hoc analyses with a Bonferroni correction. See Table 2 and Fig. 1 for means and standard errors. While there was no difference in the number of inflected forms provided by adults (*M* = 1.73) compared to children (*M* = 1.84), *F* < 1, participants supplied more inflected forms in their oral stories (*M* = 2.25) compared to their written stories (*M* = 1.32), *F*(1, 116) = 64.07, *MSe* = 3.14, *p* < .001, η^2^ = .05. There was a main effect of form as well, *F*(3, 348) = 57.82, *MSe* = 3.54, *p* < .001, η^2^ = .15. Participants more frequently used –*ing* (*M* = 2.86), followed by plurals (*M* = 1.87) and –*ed* (*M* = 1.82), with no difference between those two forms, and added –*s* to verbs (*M* = .59) the least frequently.

While there were significant two-way interactions between Age and Form, *F*(3, 348) = 11.91, *MSe* = 3.54, *p* < .001, η^2^ = .03 and Task and Form, *F*(3, 348) = 6.86, *MSe* = 2.59, *p* < .001, η^2^ = .01, these interactions were qualified by a significant three-way interaction between Age, Task and Form, *F*(3, 348) = 3.33, *MSe* = 2.59, *p* = .02, η^2^ = .01. We conducted follow-up post hoc tests to determine differences between adults and children for the different forms and different tasks. For both oral and written tasks, children produced more –*ed* verbs than adults, *t*(116) = 3.12, *p* = .002; *t*(124) = 4.94, *p* < .001, respectively. Adults provided more oral –*s* verb forms than children, *t*(116) = 3.17, *p* = .002, and more –*ing* forms in their written stories than children, *t*(124) = 2.65, *p* = .009. There were no other differences between adults and children for the inflected forms.

#### Frequency of use: all forms

**Fig. 2:**
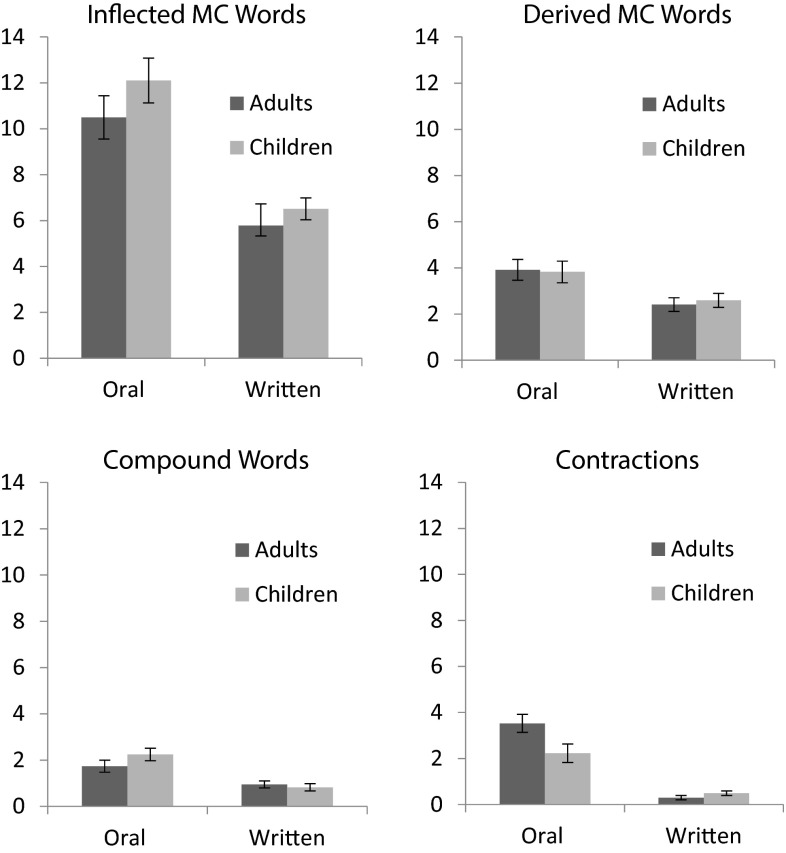
Mean (+/−SEM) number of MC words offered by adults and children across task

We counted the number of MC words that participants used in their stories, and used this score as a dependent measure. Since we wanted to compare across different types of MC words, we summed the various forms for inflected words to create a total inflected category,^3^ and then had derived, compound, and contractions form categories. We subjected these scores to a 2 (Age: Adults vs. Children) × 2 (Task: Oral vs. Written) × 4 (Form: Inflected, Derived, Compound, or Contraction) mixed ANOVA in which Age was the between groups variable and Task and Form were the repeated measures variables. See Fig. 2 for means and standard errors. Adults (*M* = 3.63) did not use more MC words in their stories compared to children (*M* = 3.85), *F* < 1. Participants produced more MC words for oral stories (*M* = 5.00) compared to written stories (*M* = 2.48), *F*(1, 116) = 85.04, *MSe* = 17.69, *p* < .001, η^2^ = .07. There were differences in the types of MC words that were used, *F*(3, 348) = 281.02, *MSe* = 9.75, *p* < .001, η^2^ = .37. Participants produced inflected (*M* = 8.72) words more frequently compared to derived words (*M* = 3.19), and those word types were produced more frequently than either compounds (*M* = 1.44) or contractions (*M* = 1.64). There was no difference between compounds and contractions.

There was a significant two-way interaction between Age and Form, *F*(3, 348) = 3.07, *MSe* = 9.75, *p* = .03, η^2^ = .01. For adults, there were significant differences among all four different MC forms, *F*(3, 180) = 126.30, *MSe* = 4.63, *p* < .001, η^2^ = .67. Adults provided more inflected forms (*M* = 8.14) than derived forms (*M* = 3.16), followed by contractions (*M* = 1.91), and finally they produced the fewest compounds (*M* = 1.34). Children also had differences among the forms they produced, *F*(3, 168) = 154.35, *MSe* = 5.14, *p* < .001, η^2^ = .73, but the pattern was different. Similar to the adults, they produced more inflected forms (*M* = 9.31) than derived forms (*M* = 3.21), followed by contractions (*M* = 1.36) and compounds (*M* = 1.54), with no differences between the latter two. There was also a significant interaction between Task and Form, *F*(3, 348) = 22.88, *MSe* = 8.80, *p* < .001, η^2^ = .03. While all of the differences comparing each form across oral and written tasks were significant, the mean difference was greater for the inflected forms (mean difference = 5.14, *t*(117) = 7.36, *p* < .001), followed by contractions (mean difference = 2.51, *t*(117) = 8.68, *p* < .001), and then there were similar mean differences for the derived forms (mean difference = 1.37, *t*(117) = 3.76, *p* < .001) and compounds (mean difference = 1.09, *t*(117) = 5.13, *p* < .001). The three-way interaction among age, form, and task was not significant, *F*(3, 348) = 1.95, *MSe* = 8.80, *p* = .12, η^2^ = .0.

### Differences on literacy measures

We also wanted to examine differences between the adults and children for other literacy measures. Thus, we assessed performance on morphological awareness, spelling, and vocabulary. We conducted *t* tests using age group as the independent variable for each of the literacy measures. There were three tasks that measured morphological awareness, so we created a composite score, which had good reliability, Cronbach’s α = .79. Across all three literacy task types, adults performed better than children: morphological awareness, *t*(124) = 5.91, *p* < .001, (adults and children, respectively, *M* = 50.43, *M* = 30.00); spelling, *t*(124) = 2.48, *p* < .02, (adults and children, respectively, *M* = 31.56, *M* = 28.27; grade equivalency scores, respectively, *M* = 5.41, *M* = 4.29); and vocabulary, *t*(124) = 5.03, *p* < .001, (adults and children, respectively, *M* = 147.38, *M* = 129.25; grade equivalency scores, respectively, *M* = 7.96, *M* = 4.94). In summary, the literacy measures tell a much more consistent story than the use of MC words in stories. Adults always outperformed children, even though these two groups were matched on non-word decoding skills.

### Correlation and regression analyses

We also conducted correlation and regression analyses using the literacy measures and the oral and written inflected and derived totals. As can be seen in Table 3, there was a different pattern of correlations between the adults and children for each of the tasks. For the adults, the literacy measures were more consistently correlated with the number of MC words produced for the oral task (all tasks but non-word decoding were significantly correlated), but only non-word decoding and spelling were correlated with the number of MC words in the written task. For the children, however, all of the literacy measures were correlated with the number MC words produced in the written task, while only vocabulary was correlated with the number of MC words produced in the oral tasks.

**Table 3:** Correlations between literacy measures and MC for oral and written tasks

	Oral task MC	Written task MC
*Adults*		
Non-word decoding	.23	.43**
Morphological awareness	.33**	.23
Spelling	.31*	.45**
Vocabulary	.28*	.07
*Children*		
Non-word decoding	.12	.47**
Morphological awareness	.18	.34**
Spelling	.15	.41**
Vocabulary	.30*	.28*

* *p* ≤ .05; ** *p* ≤ .01

For the regression analyses, the total number of all MC words for each task (oral and written) were used as the predictor variables, while the literacy measures (non-word decoding, morphological awareness, spelling, and vocabulary) were each used as outcome variables. See Table 4 for *R* ^2^, *β*, and *t*-values. When non-word decoding was the outcome variable, the use of MC words explained a significant amount of the variance for both the adults, *R* ^2^ = .20, *F*(2,58) = 7.33, *MSe* = 46.53, *p* = .001, and the children, *R* ^2^ = .22, *F*(2,54) = 7.39, *MSe* = 39.90, *p* = .001. As can be seen in Table 4, for both groups of participants, the MC written total variable was a unique predictor of non-word decoding. MC totals also explained a significant portion of the variance in morphological awareness for both the adults, *R* ^2^ = .13, *F*(2,58) = 4.37, *MSe* = 395.40, *p* = .02, and the children, *R* ^2^ = .14, *F*(2,54) = 4.23, *MSe* = 280.46, *p* = .02. For the adults, the oral MC total was a unique predictor, while written MC total was the unique predictor for the children. The MC totals explained a significant portion of the variance for spelling for both adults, *R* ^2^ = .25, *F*(2,58) = 9.82, *MSe* = 43.29, *p* < .001, and children, *R* ^2^ = .19, *F*(2,54) = 6.28, *MSe* = 45.14, *p* = .004. For both adults and children, the written MC totals were unique predictors in the model. For vocabulary, MC totals did not explain a significant portion of the variance for the adults, *R* ^2^ = .07, *F*(2,58) = 2.38, *MSe* = 450.28, *p* = .10, but they did for the children, *R* ^2^ = .14, *F*(2,54) = 4.43, *MSe* = 329.44, *p* = .02; however, neither of the variables were unique predictors. For the most part, the regression analyses were consistent across participant groups. The use of MC words explained a significant portion of the variance in non-word decoding, morphological awareness, and spelling abilities for both groups. Written MC totals were more consistently unique predictors in these analyses.

**Table 4:** Regression analyses with literacy measures as outcome variable and MC for oral and written tasks as predictor variables

	*R* ^2^	*β*	*t*
*Adults*			
Non-word decoding	.20**		
MC oral task		.01	.77
MC written task		.42	3.28**
Morphological awareness	.13*		
MC oral task		.28	2.15*
MC written task		.16	1.19
Spelling	.25***		
MC oral task		.18	1.50
MC written task		.41	3.43**
Vocabulary	.08		
MC oral task		.28	2.05*
MC written task		.002	.15
*Children*			
Non-word decoding	.22**		
MC oral task		.01	.07
MC written task		.46	3.71***
Morphological awareness	.14*		
MC oral task		.10	.77
MC written task		.33	2.54*
Spelling	.19**		
MC oral task		.05	.43
MC written task		.42	3.32**
Vocabulary	.14*		
MC oral task		.25	1.89
MC written task		.23	1.79

* *p* ≤ .05; ** *p* ≤ .01; *** *p* < .001

## Discussion

The purpose of the current study was to examine the differences associated with incorporating MC words in the stories of both adults with low literacy skills who were enrolled in ABE programs and children (grades 1, 2, 3, and 5) who were matched with the adults on decoding ability. We assessed both oral and written stories to determine if the written narratives, which are cognitively more demanding, would mask some of the production of these complex words. In addition, we collected data on other tasks that reflect reading and writing development. We have several interesting findings. Both adults and children were more likely to produce MC words in their oral stories compared to their written ones. While children were much more skilled at using –*ed* forms to produce past tense verbs than adults, adults were more likely to add –*s* to a verb and to produce contractions. Children and adults were comparable in pluralizing words, adding –*ing* to verbs, and producing derived MC words. For the literacy measures, interestingly, adults always outperformed children, even though these two groups were matched on non-word decoding skills. Thus, while adults outperformed the children in terms of morphological awareness, spelling, and vocabulary, those skills did not seem to aid in the production of explicitly applying morphological knowledge in the story-telling tasks. Finally, regression analyses were conducted in which MC words were predictors for the literacy measures. The production of MC words explained significant variance in non-word decoding, morphological awareness, and spelling. However, for vocabulary skills, the production of MC words was a significant predictor only for the children, and not the adults.

The adults with low literacy skills and children appear most competent in pluralizing and adding –*ing* to verbs. While those forms were most prevalent in the oral stories, the majority of the participants incorporated them into their written stories as well. A much different story was found for the use of –*ed*. Most of the children represented the past tense in their oral and written stories, by using the –*ed* marker. Fewer than half of the adults, on the other hand, ever used a past tense verb in either their oral or written stories. Carlisle (1996) found that 95 % of her third grade sample used the past tense at least once, yet our adults performed far below that level. Children clearly outperformed the adults on using past tense verbs in their stories. Why might the past tense be so difficult for these adults? The importance of rules regarding morphology and spelling contradicts some of the phoneme-grapheme correspondences when alternative pronunciations exist for word endings that are spelled the same way. For example, the past tense inflection, -*ed*, presents a challenge because *kissed* (*/t/*sound), *killed* (*/d/*sound), and *waited* (/-*ə* *d/*sound) are all spelled the same, but pronounced differently (Carlisle, 2010). Thus, the inconsistent correspondence between spelling and sound may contribute to the difficulty adults with low literacy skills have with using the past tense.

While neither group demonstrated proficiency for adding –*s* to verbs (i.e., third person singular present tense), the adults did outperform children on this dimension. That is, adults used this verb form 53 and 33 % in their oral and written stories, respectively, while children used them only 16 and 10 % of the time. Thus, it was not the case that children always outperformed adults with the inflected forms.

Both adults and children demonstrated some skill in using derived forms in both their oral and written stories. While most of the participants incorporated at least one derived word into their stories, they used far fewer derived words than inflected ones. Since knowledge and use of inflected morphemes develops earlier, this finding is not surprising, however, it is interesting that the use of derived words by both groups is so much lower compared to the use of inflected words. The reduced rate of the production of derived words is consistent with the work of Green et al. (2003). They found that by the end of 4th grade, only 57 % of their participants were using derived forms in their writing. Thus, both children and adults with low literacy skills need work on producing derived words in their stories.

Since adults outperformed children on the morphological awareness tasks, it is surprising that we did not see the implicit awareness skills translating to explicit production. That is, based on the morphological awareness tasks, we would have predicted that adults would have been able to call on this knowledge to produce more MC words than children in their stories. If adult learners have this skill, why are they less likely to use MC words in their written and oral stories? One explanation could have to do with the nature of the two types of tasks. In the morphological awareness tasks, the format is much more constrained than in the storytelling tasks. The awareness tasks explicitly encourage participants to tap into a specific skill set, exposing only a narrow view of their abilities and representations of that one skill. However, a more free-form task such as storytelling does not require the participant to tap into any one particular skill. Instead, the participant can rely upon the skills with which she/he is most familiar or confident. For adults with low literacy skills, telling a story—either written or oral—may incorporate skills such as working memory, vocabulary, and/or context more than morphological awareness.

One other potential explanation that we must consider is the experiences adult learners have with writing and story-telling are markedly different than the experiences of young learners. Schwertman and Corey (1989) argue that negative experiences with education, low self-esteem, and less risk taking in writing are hallmarks of adult learners. Whereas contemporary elementary school-aged children are encouraged to take risks in their writing—often at the expense of spelling or vocabulary errors (Viise, 1996), adults may not have this similar experience. Combined with a lower sense of self-efficacy in their writing and verbal abilities, this may lead adult learners to err on the side of caution and not tap into some resources such as morphological awareness. For example, an adult learner who is less confident in her ability to correctly inflect the verb *run* with an -*ing* ending to articulate the idea “The boy is running” may avoid conjugating the verb altogether (e.g. “The boy run.”) or rely on other skills to avoid having to use that inflection (“The boy ran”). These conclusions are entirely speculative as the present study investigated only the use of MC words in written and oral stories. However, further research should also consider the types of errors made within these stories to explain why adult learners underutilize MC words in their stories—even though they appear to have a comparably reliable understanding of morphology in English language usage. Additionally, if problems associated with spelling are the reason the adults fail to show development in using MC words in their writing, perhaps future directions should investigate the implementation of morphological interventions to spark developmental growth for the adults with low literacy skills. In a recent meta-analysis, Goodwin and Ahn (2013) found that morphological interventions produced a moderate effect size for improvements in children’s spelling. Perhaps these interventions would be effective in an adult literacy classroom as well.

One serious limitation associated with the present study is that the skill range associated with our participants was quite large, but since our sample was relatively modest, we did not have enough power to examine differences across skill-level. We also have some variation in native language abilities in both samples, and this, too, could have an influence on morphological awareness and word production abilities. Unfortunately, due to issues of power, we could not examine the impact of native language skills on MC word production. In addition, ideally, one would be able to examine developmental changes in these abilities over time. However, implementing a longitudinal design with children is much more feasible than using one with adult learners. A child’s job is to go to school, so children can more or less reliably be found at their school for the assessments. Adult lives are more complicated. They are not embedded in traditional educational contexts, and they often have to balance school, work responsibilities, and parenting. Attendance can be sporadic, and the attrition rate for adults from ABE programs is sizeable (e.g., Alamprese, MacArthur, Price, & Knight, 2011; Sabatini, Shore, Holtzman, & Scarborough, 2011). Thus, using a longitudinal approach for this research question seems impractical. An additional limitation to note is that the adults were tested in one session rather than across 2 days like the children; therefore, time sampling error may be an issue for the adult participants.

In summary, adults with low literacy skills are different than younger early readers in several important ways, including their ability to rely upon context and orthographic knowledge to aid in comprehension (Thompkins & Binder, 2003), and may use these skills to compensate for phonological decoding deficits. The findings of the current study extend our understanding of the adult learners’ cognitive profile, as they suggest that adult learners have better vocabulary knowledge, spelling ability, and morphological awareness than younger readers matched on decoding skill, yet children show more skill in using MC words in their stories than the adults. Previous research has established that adult learners are sensitive to the morphological structure of words (Tighe & Binder, 2014) during word recognition and reading comprehension. However, more work is needed to understand the relationship between implicit morphological awareness and translating that knowledge into explicitly representing that information in both oral and written language.

## Footnotes

1 Pilot testing revealed 5 min of written storytelling produced a similar word count as 1½ min of oral storytelling, which is why these procedural times were chosen.

2 We didn’t include –*en* forms since many cells had a mean and standard deviation of 0 for that form.

3 For this analysis we did include *–en* forms.
